# Phenotypic Switch in Blood: Effects of Pro-Inflammatory Cytokines on Breast Cancer Cell Aggregation and Adhesion

**DOI:** 10.1371/journal.pone.0054959

**Published:** 2013-01-23

**Authors:** Yue Geng, Siddarth Chandrasekaran, Jong-Wei Hsu, Mishka Gidwani, Andrew D. Hughes, Michael R. King

**Affiliations:** Department of Biomedical Engineering, Cornell University, Ithaca, New York, United States of America; University of California, San Diego, United States of America

## Abstract

Hematogeneous metastasis can occur via a cascade of circulating tumor cell adhesion events to the endothelial lining of the vasculature, i.e. the metastatic cascade. Interestingly, the pro-inflammatory cytokines IL-6 and TNF-α, which play an important role in potentiating the inflammatory cascade, are significantly elevated in metastatic breast cancer (BCa) patients. Despite their high metastatic potential, human breast carcinoma cells MDA-MB-231 lack interactions with E-selectin functionalized surfaces under physiological shear stresses. We hypothesized that human plasma, 3-D tumor spheroid culture, and cytokine-supplemented culture media could induce a phenotypic switch that allows BCa cells to interact with E-selectin coated surfaces under physiological flow. Flow cytometry, immunofluorescence imaging, and flow-based cell adhesion assay were utilized to investigate the phenotypic changes of MDA-MB-231 cells with various treatments. Our results indicate that plasma, IL-6, and TNF-α promote breast cancer cell growth as aggregates and induce adhesive recruitment of BCa cells on E-selectin coated surfaces under flow. 3-D tumor spheroid culture exhibits the most significant increases in the interactions between BCa and E-selectin coated surfaces by upregulating CD44V4 and sLe^x^ expression. Furthermore, we show that IL-6 and TNF-α concentrations in blood may regulate the recruitment of BCa cells to the inflamed endothelium. Finally, we propose a mechanism that could explain the invasiveness of ‘triple-negative’ breast cancer cell line MDA-MB-231 via a positive feedback loop of IL-6 secretion and maintenance. Taken together, our results suggest that therapeutic approaches targeting cytokine receptors and adhesion molecules on cancer cells may potentially reduce metastatic load and improve current cancer treatments.

## Introduction

Cancer mortality is predominantly caused by the dissemination of cancer cells from the primary tumor to distant organs where secondary sites are formed via a metastatic progression. Breast cancer, one of the most diagnosed forms of cancer, still causes high mortality rates due to the emergence of invasive, therapy-resistant cancer cells [1]. There are conflicting ideas on the nature of these invasive cells, from their population size within the primary tumor [2], [3] to their methods of dissemination (lymphatic [4] or hematogeous [5]) and the process of metastasis within blood vessels [6], [7], [8].

One mode of cancer metastasis is through the bloodstream, which involves the escape of cancer cells from the primary tumor site into the circulatory system via intravasation. Circulating tumor cells (CTCs) can then interact and adhere to the endothelial lining of the vasculature through a series of receptor-mediated events, commonly referred to as the metastatic adhesion cascade. This cascade mimics the leukocyte adhesion cascade (reviewed in [5]) where the initial contact between cancer cells and the endothelium is facilitated by a family of endothelial adhesion molecules called selectins [9], [10]. Firm adhesion of CTCs on the endothelium then allow extravasation and subsequent secondary tumor site formation.

There are several factors in the blood that are secreted by lymphocytes and macrophages known to facilitate the metastatic progression and potentially interact with CTCs within the bloodstream. In particular, interleukin-6 (IL-6) and tumor necrosis factor-alpha (TNF-α) have been reported to be elevated in the blood serum of patients diagnosed with advanced stage breast tumor and correlate with an increased number and size of metastatic sites [11], [12]. IL-6 and TNF-α have been shown to promote the growth and invasiveness of colon and prostate cancer epithelial cells *in vitro* and *in vivo* [13], [14]. Studies have also shown that cancer cell chemokine receptors also play a role in determining the destination of metastases [15].

MDA-MB-231 is a well characterized metastatic breast cancer cell line isolated from pleural effusion of a patient with adenocarcinoma suffering from widespread metastasis, and is known as a ‘basal’ or ‘triple-negative’ cell line with stem cell-like or post-Epithelial-Mesenchymal Transition (EMT) features and fails to express estrogen receptors, progesterone receptors or human epidermal growth factor receptor 2 (HER2). MDA-MB-231 cells have been previously reported to express E-selectin ligands such as CD44V4 as well as selectin-binding moieties such as sialyl lewis x (sLe^x^) [16], [17]. Despite their expression of functional E-selectin ligands, we have observed that MDA-MB-231 cells do not adhesively interact with E-selectin coated microtube surfaces under flow, as reported here. In the present study, we investigated the effect of plasma, cytokine treatments, and 3-D spheroid culture conditions on altering the adhesion phenotype of MDA-MB-231 cells on E-selectin functionalized microtubes. We hypothesized that human plasma, tumor spheroid culture, and exposure to pro-inflammatory cytokines such as IL-6 and TNF-α could induce robust interactions between metastatic breast cancer cells in circulation and the inflamed endothelium. The findings of this study suggest new therapeutic approaches targeting specific cytokine(s) and their receptor(s) to help prevent the metastatic progression of breast cancer cells in transit.

## Results

### Blood plasma triggers an adhesive phenotypic switch of breast cancer (BCa) cells on E-selectin coated surfaces under flow

**Figure 1:**
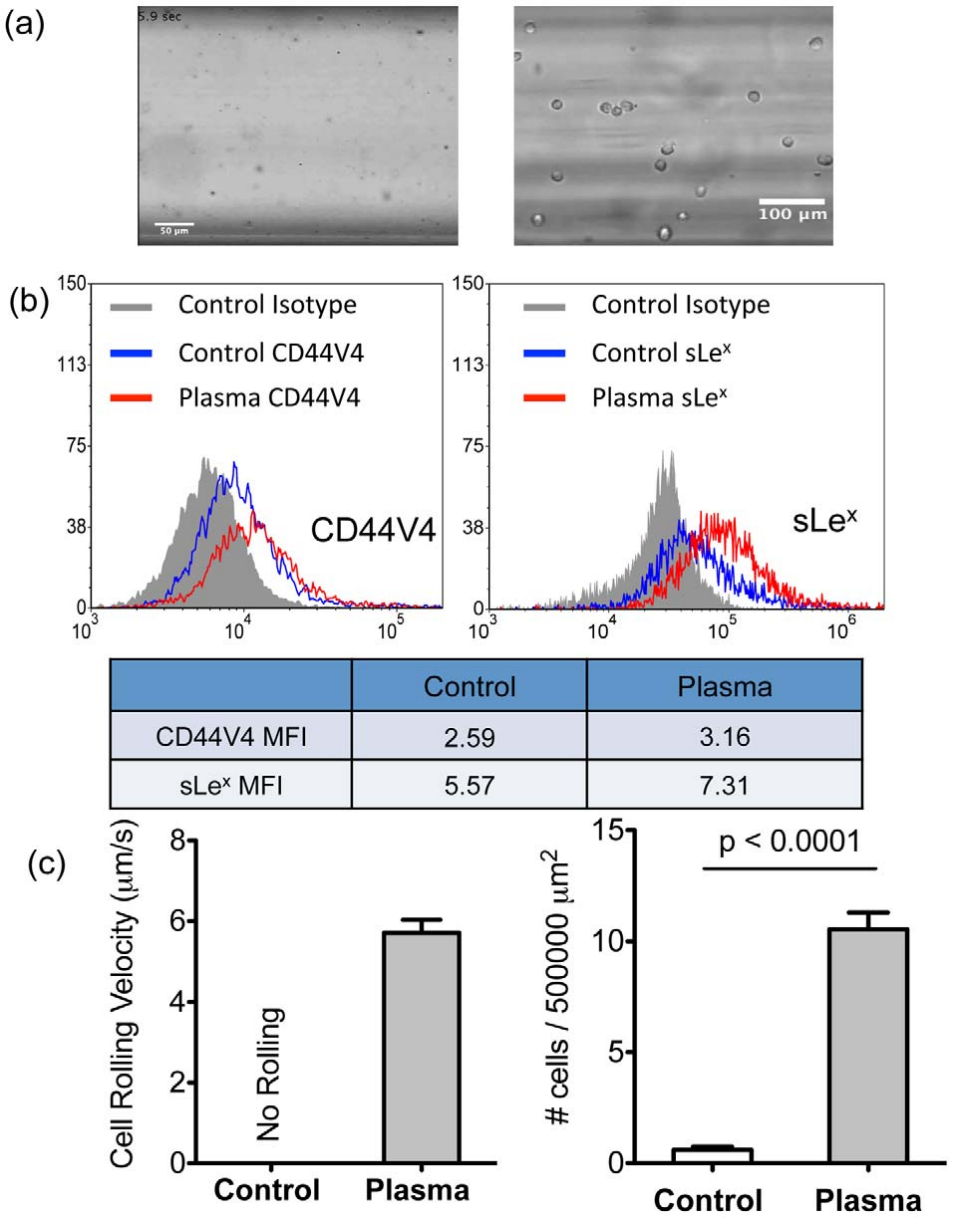
Plasma triggers an adhesive phenotypic switch of breast cancer (BCa) cells on E-selectin coated surface under flow. (a) Left: Untreated MDA-MB-231 cells (2-D culture with regular medium) show no interactions with the E-selectin coated surfaces under flow. Right: Cells establish stable rolling on E-selectin coated surface after 48 h plasma treatment. (b) Flow cytometry results of CD44V4 (left) and sLe^x^ (right) expression on untreated and plasma treated MDA-MB-231 cell surfaces. Mean fluorescence intensity ratio of sample over isotype has been included. (c) Left: Average rolling velocity of untreated (control) and plasma treated MDA-MB-231 cells (n = 35 cells). Shear stress of 1 dyn/cm^2^ was used. Right: Average numbers of cells interacting with the E-selectin coated surface per 5×10^5^ μm^2^ of untreated (n = 30 frames) and plasma treated (n = 36 frames) MDA-MB-231 cells. Bars represent mean ± SEM. P<0.0001, two-tailed unpaired student t-test.

When grown in 2-D monolayers with culture media, highly metastatic MDA-MB-231 breast cancer cells showed no adhesive interactions with E-selectin coated surfaces under flow conditions (Figure 1a) despite the expression of the E-selectin ligand CD44V4 and the binding moiety sLe^x^ (Figure 1b). This is contradictory to its reported high metastatic potential from *in vivo* studies where MDA-MB-231 cells were found to efficiently metastasize to distant organs through the bloodstream [18], where selectin-mediated tethering and rolling events have been shown to play important roles, as reviewed in [19].

A more physiological method of culturing cancer cells is presented where human blood plasma is used in combination with culture media. The plasma treatment was found to switch the MDA-MB-231 cells from non-interacting to adhesive (Figure 1a) where conventionally cultured MDA-MB-231 cells (control) exhibited no rolling cells on an E-selectin coated surface and plasma cultured cells exhibited strong rolling behavior with rolling velocities of 5.72±0.32 μm and a 10-fold increase of cell flux compared to control cells (Figure 1c). It was found that sLe^x^ expression was upregulated on cells cultured in plasma compared to control cells (Figure 1b), which may contribute to the resulting adhesive phenotype.

### Pro-inflammatory cytokines IL-6 and TNF-α induce adhesive recruitment of BCa cells and is blocked by the anti-inflammatory drug Metformin

**Figure 2:**
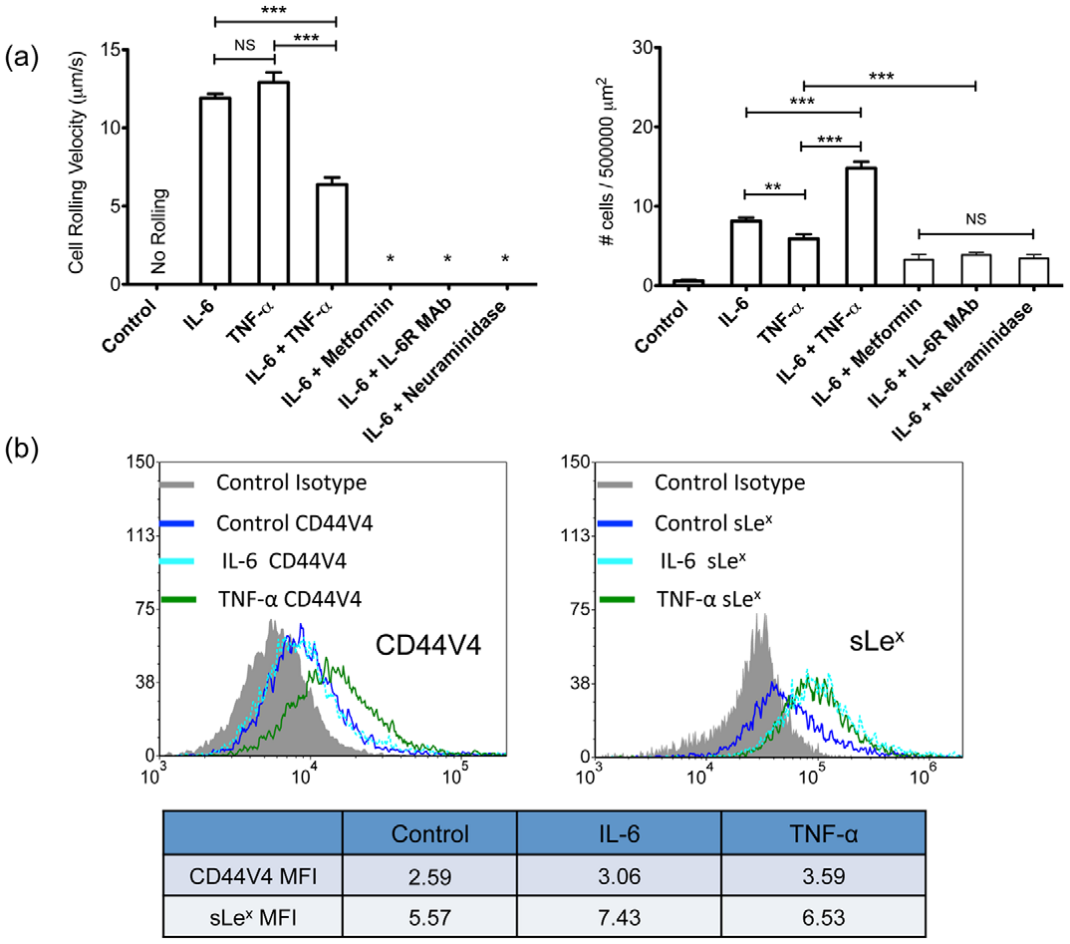
Pro-inflammatory cytokines IL-6 and TNF-α also induce adhesive recruitment of BCa cells and is blocked by the anti-inflammatory drug Metformin. (a) Average rolling velocity (left) and average number of MDA-MB-231 cells interacting with E-selectin coated surfaces (right) after treatments including 5 ng/mL of IL-6 (n = 71), 5 ng/mL TNF-α (n = 48), IL-6+ TNF-α (5 ng/mL each), 5 ng/mL of IL-6 with 0.1 mmol Metformin, 5 ng/mL of IL-6 with 1 μg/mL anti-IL-6R MAb, 5 ng/mL of IL-6 with neuraminidase treatment prior to rolling experiment. For conditions where MDA-MB-231 were treated with IL-6 with MAb, Metformin, or neuraminidase (after IL-6 treatment), there were insufficient numbers of cells found on the E-selectin coated surface for velocity analysis. Bars represent mean ± SEM. P<0.0001, two-tailed unpaired student t-test. Average number of MDA-MB-231 cells from control experiment was found to be significantly less than all other conditions (P<0.0001). (b) Flow cytometry measurements of CD44V4 (left) and sLe^x^ (right) expression on untreated, IL-6 (5 ng/mL), and TNF-α (5 ng/mL) treated MDA-MB-231 cell surfaces. Mean fluorescence intensity ratio of sample over isotype has been included.

Pro-inflammatory cytokines have been linked to poor prognosis in metastatic breast cancer patients with elevated IL-6 and TNF-α cytokine in their blood plasma [11], [12]. Similar to culture in plasma, MDA-MB-231 cells cultured in IL-6 and TNF-α supplemented media also induced the adhesive phenotypic switch resulting in cell rolling velocities of 11.90±0.28 μm/s and 14.22±0.85 μm/s for IL-6 and TNF-α conditions, respectively, twice that of plasma and the combination of IL-6 and TNF-α treated cell rolling velocities (Figure 2a). Treatment with IL-6 and TNF-α at physiologically relevant concentrations such as 25 pg/mL also induced rolling on E-selectin coated microtubes at rolling velocities of 15.43±1.31 μm/s and 17.03±2.21 μm/s, respectively. Furthermore, whereas TNF-α upregulated both CD44V4 and sLe^x^ expression compared to control cells, IL-6 only upregulated sLe^x^ (Figure 2b) suggesting the importance of increased sLe^x^ moieties. Blocking the IL-6 receptor with anti-IL-6R monoclonal antibody effectively hindered the adhesive phenotype resulting in nearly abolished cell flux on the E-selectin surfaces (Figure 2a). Treating cells with an isotype control for the monoclonal antibody did not affect the IL-6 induced MDA-MB-231 cell rolling on E-selectin coated microtubes (data not shown). In some experiments, IL-6 treated MDA-MB-231 cells were incubated with neuraminidase for 1 hr to cleave sLe^x^ from the cell surface. Interactions between MDA-MB-231 cells and the E-selectin coated surfaces were abolished after neuraminidase treatment, indicating that the rolling behavior of MDA-MB-231 cells is E-selectin: sLe^x^ dependent (Figure 2a). Interestingly, the anti-inflammatory drug Metformin (1,1-dimethylbiguanide hydrochloride), a frequently used drug to treat type 2 diabetes, showed similar effects to the IL-6R antibody. Metformin has been shown to target Stat3 and induce apoptosis in triple-negative breast cancers as well as significantly decrease serum IL-6 level in breast cancer patients [21], [22].

### Plasma, IL-6, and TNF-α promote breast cancer cell growth as aggregates

**Figure 3:**
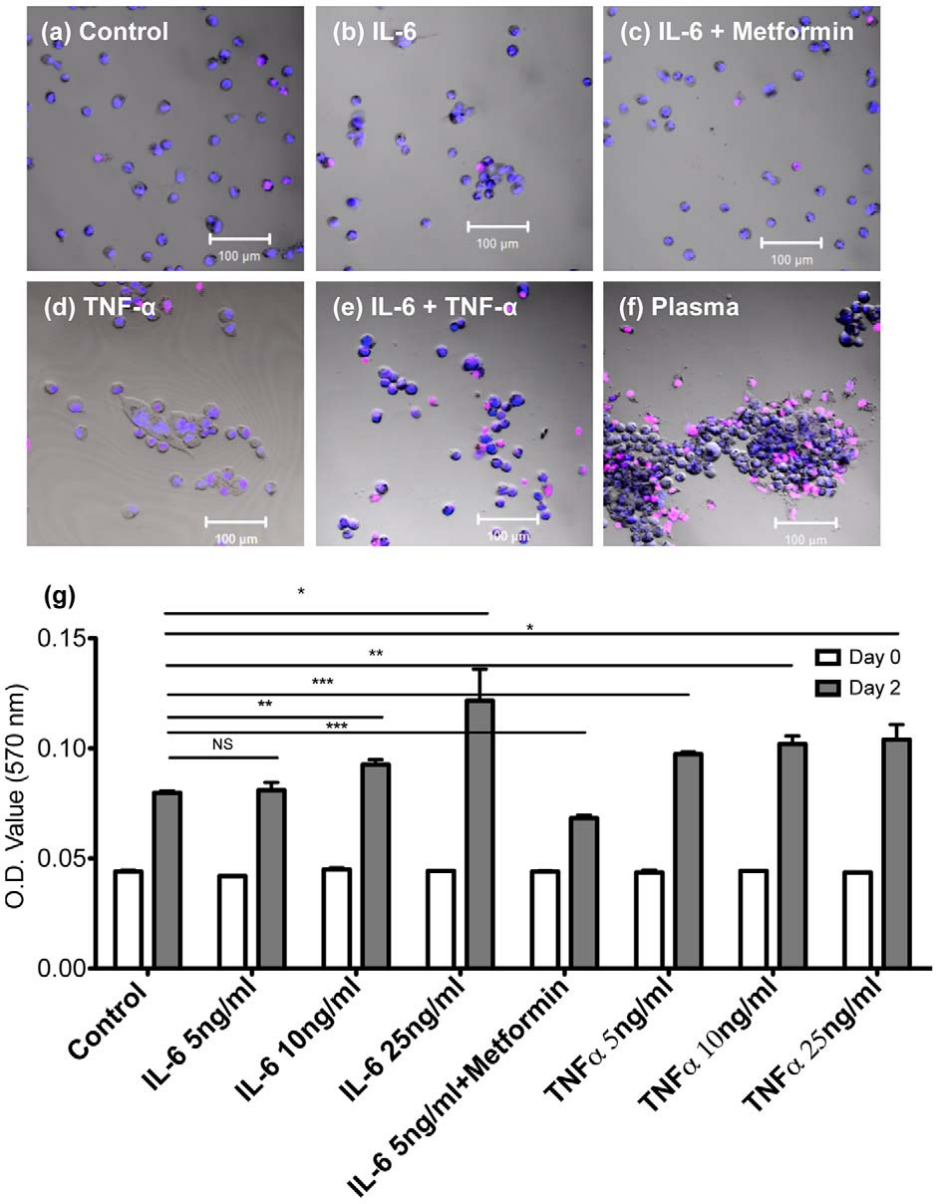
Plasma, IL-6, and TNF-α promote breast cancer cell growth as aggregates. Morphology and viability of MDA-MB-231 cells cultured in (a) regular medium, (b) IL-6 (5 ng/mL) spiked medium, (c) IL-6 (5 ng/mL) and 0.1 mmol Metformin added medium, (d) TNF-α (5 ng/mL) spiked medium, (e) IL-6 and TNF-α (5 ng/mL each) spiked medium, and (f) healthy donor plasma. A blue fluorescent cell-permeable nucleic acid dye (for live cell staining) and a red fluorescent cell-impermeable nucleic dye (for dead cell staining) were used to visualize the live and dead populations. (g) Cell proliferation results via MTT assay of control media, IL-6, TNF-α, and Metformin treated MDA-MB-231 cells.

When plated in a 24 well plate with imaging ready glass coverslip built-in wells, control cells were found to grow as monolayers with very little occurrence of aggregation (Figure 3a). Monitoring the cell proliferation and viability under various cytokine treated culture media conditions demonstrated that not only do MDA-MB-231 cells proliferate at an increased rate in the presence of IL-6 and TNF-α (Figure 3g), an increased number of aggregates (Figures 3b, d, and e) was observed. A similar and enhanced effect was observed in human plasma (Figure 3f) where the large degree of aggregation is reminiscent of tumor spheroids. Therefore, the cell growth and formation as aggregates may cause the adhesive phenotypic switch, especially considering that MDA-MB-231 cells grown in IL-6 and Metformin formed a negligible number of aggregates (similar to control cells, Figure 3c). MTT assay also indicated increased cell proliferation when MDA-MB-231 cells were treated with high concentrations of IL-6 and TNF-α (Figure 3g).

### 3-D tumor spheroid culture increases the interactions between BCa cells and E-selectin coated surfaces by upregulating CD44V4 and sLe^x^ expression

**Figure 4:**
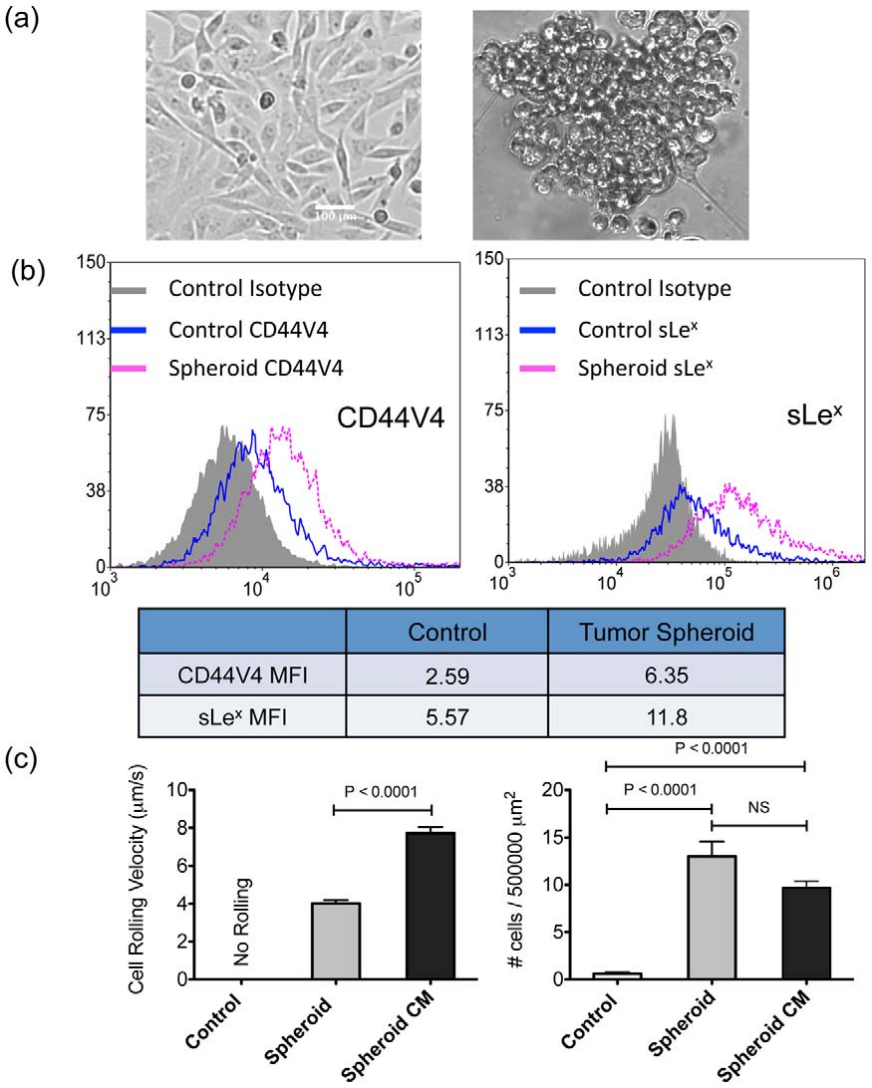
3-D tumor spheroid culture further increases the interactions between BCa cells and E-selectin coated surfaces by upregulating CD44V4 and sLe^x^ expression. (a) Micrographs of MDA-MB-231 cells cultured in 2-D monolayer (left) and 3-D spheroid on PDMS (right). (b) Flow cytometry results of CD44V4 (left) and sLe^x^ (right) expression on untreated (2-D monolayer grown) and tumor spheroid cultured MDA-MB-231 cells. Tumor spheroid cultured cells were treated with enzyme free dissociation buffer prior to experiments to obtain mostly single cell populations. Mean fluorescence intensity ratio of sample over isotype has been included. (c) Average rolling velocity (left) and average number of MDA-MB-231 cells found interacting with E-selectin coated surfaces (right) cultured in medium (untreated), 3-D spheroid, and spheroid conditioned medium. Bars represent mean ± SEM. Two-tailed unpaired student t-test was used. (d) CD44 mRNA expression of MDA-MB-231 cells that are cultured in control, IL-6, TNF-α, tumor spheroid, and plasma conditions, respectively, measured via qPCR.

Similar to elevated cytokine concentrations, the increased occurrence of tumor spheroids and circulating tumor microemboli has been linked to poor prognosis and increased metastatic potential [23], [24]. Culturing MDA-MB-231 cells as 3-D tumor spheroids in conventional culture media (Figure 4a) had an even greater effect on cell adhesiveness to an E-selectin coated surface. Comparable to plasma and cytokine treated conditions, cells grown as spheroids also showed significantly increased CD44V4 and sLe^x^ expression (Figure 4b) while inducing more stable rolling on E-selectin coated surfaces under flow. Moreover, as shown in Figure 4c, MDA-MB-231 cells grown in tumor spheroid conditioned media also demonstrated the adhesive phenotypic switch with an average rolling velocity of 7.72±0.32 μm/s, although significantly faster than MDA-MB-231 cells grown in 3-D spheroids (4.02±0.17 μm/s). mRNA expression of CD44 was also measured for all conditions (IL-6, TNF-α, tumor spheroid, and plasma) via real time quantitative PCR (qPCR). As shown in Figure 4d, all conditions resulted in upregulation of CD44 mRNA expression compared to control.

### IL-6 and TNF-α concentrations in blood may regulate the recruitment of BCa cells to the inflamed endothelium

**Figure 5:**
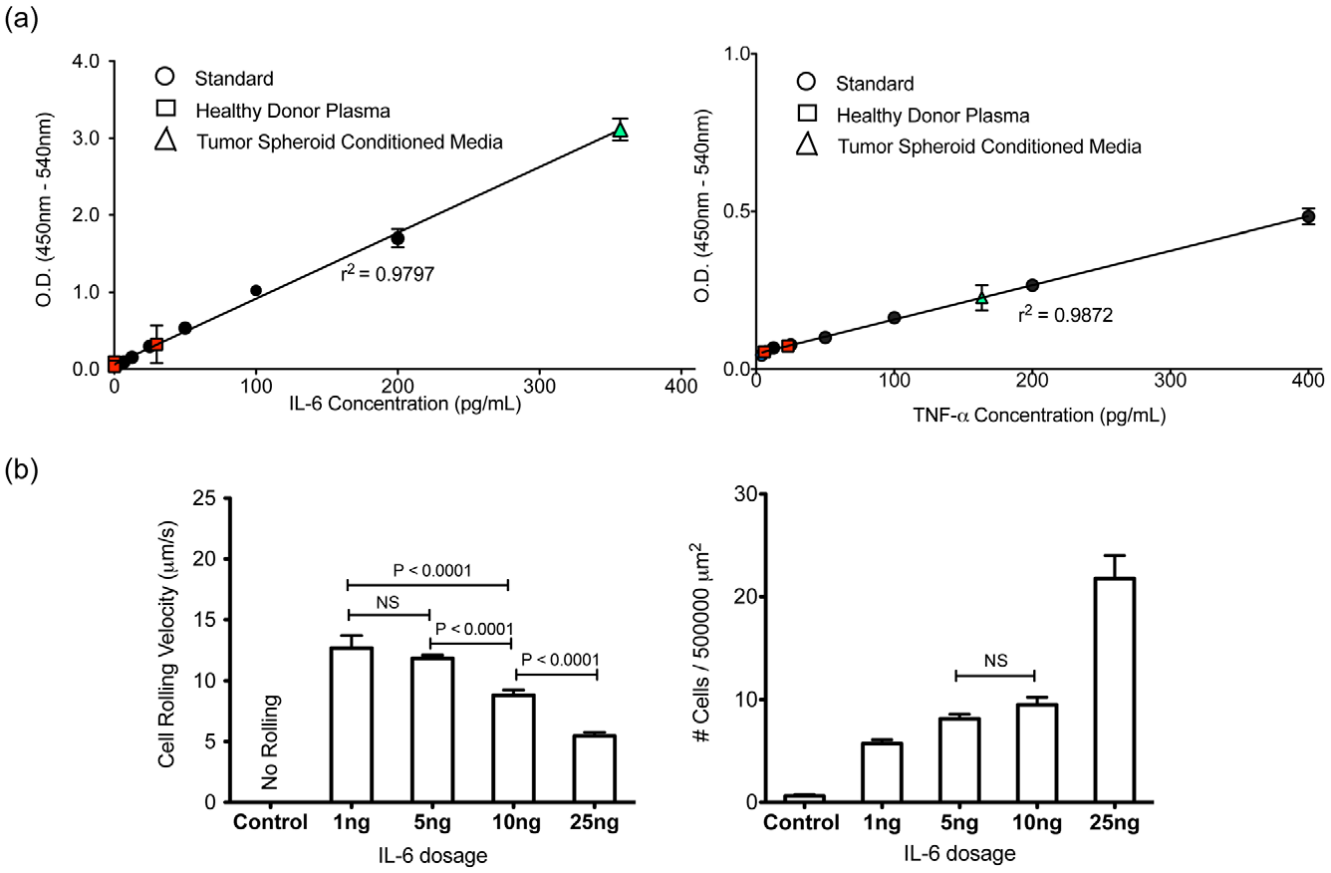
IL-6 and TNF-α concentrations in blood can regulate the recruitment of BCa cells to the inflamed endothelium. (a) Calculated concentrations of IL-6 (left) and TNF-α (right) of healthy female donor plasma and tumor spheroid conditioned media on ELISA standard curves. IL-6 concentration in the tumor spheroid conditioned media was diluted with equal volume of culture media to remain in the detectable range (0–200 pg/mL) of the ELISA kit. (b) Average rolling velocities (left) and average numbers of MDA cells found interacting with E-selectin coated surfaces (right) from untreated conditions and culture media spiked with a range of IL-6 concentration including 1, 5, 10, and 25 ng/mL. For the cell number analysis, all conditions are significantly different (p<0.0001) except for the 5 and 10 ng/mL treatments. Bars represent mean ± SEM. Two-tailed unpaired student t-test was used.

Interestingly, the tumor spheroid (3-D) conditioned media contained significantly higher concentrations of both IL-6 and TNF-alpha (Figure 5a) than that of healthy donor plasma. Conditioned media from 2-D culture was also assayed and the concentrations of both IL-6 and TNF-alpha were significantly lower than physiological cytokine concentrations sufficient to have an effect on the phenotype of MDA-MB-231 cells. To explore the effects of cytokine concentration on the adhesive behavior of MDA-MB-231 cells, human recombinant IL-6 was added to cell culture medium at a range of concentrations consisting of 1, 5, 10, and 25 ng/mL. MDA-MB-231 cells treated with 25 ng/mL of IL-6 demonstrated the strongest interactions with the E-selectin coated surface with a rolling velocity of 5.46±0.31 μm, comparable to the rolling velocity of plasma cultured cells (Figure 5b). Overall, MDA-MB-231 cells showed more robust interactions with E-selectin coated surfaces as the concentration of IL-6 was increased, resulting in greater numbers of cells recruited via the adhesion cascade.

## Discussion

There is compelling evidence supporting the strong interplay between various types of inflammation and cancer progression suggested by numerous clinical and epidemiological studies [25], [26]. The recruitment of tumor-associated immune cells during the inflammation that accompanies tumor progression, has been shown to promote tumor growth and contribute to angiogenesis, invasion, and metastasis [27]. Infiltration of these tumor-promoting immune cells as well as various inflammatory cytokines, particularly TNF-α and IL-6, has been found in the breast tumor microenvironment [28]. A clinical study reported elevated levels of pro-inflammatory cytokines in the serum of breast cancer patients when compared to healthy individuals [29]. As one of the well characterized ‘triple-negative’ breast cancer cell lines, MDA-MB-231 has been reported to also be mesenchymal stem cell-like [30]. Furthermore, ‘triple-negative’ breast cancers have been found to correlate with inflammatory states such as obesity and diabetes where pro-inflammatory cytokines, including IL-6, are highly expressed.

The lack of interaction between culture medium grown MDA-MB-231 cells with E-selectin coated surfaces, as observed in this study, was rather unexpected considering their reported high metastatic potential from both *in vitro* and *in vivo* studies. We have shown that human plasma, IL-6, and TNF-α supplemented culture conditions induced an adhesive phenotypic switch, allowing MDA-MB-231 cells to establish stable rolling on E-selectin coated surfaces under physiological flow. Plasma factors such as fibrinogen as well as other cytokines such as IL-1β and IFN-γ have also been shown to promote cell adhesion [31], [32], [33]. In this study, we focused on IL-6 and TNF-α due to their association with poor prognosis for breast cancer patients. The concentration of cytokines used for our in vitro experiments was based on similar studies that have investigated the effect of these cytokines on invasiveness of breast cancer cell lines [34]. CD44V4, a major E-selectin ligand for breast cancer cells, was found to be upregulated on MDA–MB-231 cells treated with TNF-α. TNF-α has been reported to increase CD44 expression on ovarian cancer cells upon the activation of the c-Jun NH_2_-terminal kinase [35] and, although not in the context of cancer, affects the glycosylation and sulfation of various glycoproteins [36]. On the other hand, IL-6 has been shown to induce a significant increase in the expression of α1, 3/4-fucosyltransferases (FUT11 (fucosyltransferase 11 gene) and FUT3) as well as the amounts of sLe^x^ and 6-sulfo-sLe^x^ epitopes in human bronchial mucosa [37]. Although CD44V4 is a major E-selectin ligand for breast cancer cells, there are other ligands such as MUC1 which play an active role in rolling and firm adhesion of breast cancer cells on inflamed endothelium, as reported in our recent study [38]. The flow cytometry results of the present study comparing control and IL-6 treated MDA-MB-231 cells suggest that IL-6 also promotes the synthesis of sLe^x^ on breast cancer cells, potentially providing more opportunity for sLe^x^: E-selectin mediated heterotypic interaction between cancer cells and the activated endothelium.

Selectin-based technology has been used to capture viable circulating tumor cells (CTCs) that can be further characterized by releasing the bound CTCs in the absence of calcium [39], [40]. Recently, E-selectin-mediated adhesive interactions were utilized inside an E-selectin coated polydimethylsiloxane (PDMS) microbubble system to capture colon CTCs under flow conditions [41]. Presently, most CTC studies have focused on the survival and metastasis of individual tumor cells in the circulation. However, emerging evidence supports the importance of circulating tumor microemboli (CTM) during cancer progression [42]. CTM represent a “collective migration” of tumor cells and have been found to exhibit higher metastatic potential than that of individual CTCs [43]. It has been proposed that these multicellular clumps of tumor cells can metastasize by becoming mechanically trapped in the smallest capillaries due to their large size [23]. We found that tumor spheroid/aggregate grown MDA-MB-231 breast cancer cells upregulate their number of E-selectin ligands and binding moieties and demonstrated the most robust rolling behavior on E-selectin coated surfaces, compared to MDA-MB-231 cells from plasma and cytokine treatments, suggesting that CTMs may efficiently escape the bloodstream via a metastatic adhesion cascade.

Highly metastatic tumor cells have been reported to exhibit increased homotypic aggregation, gaining resistance to apoptosis [24]. There is also evidence to suggest that breast cancer cells cultured as 3-D spheroids express a higher level of IL-6 mRNA [44]. A recent study by our group reported that homotypic and heterotypic interactions in breast cancer cells cultured as 3-D spheroids alter their adhesion phenotype. These interactions were shown to increase the interaction of breast cancer cells with human recombinant E-selectin [45]. Furthermore, when MDA-MB-231 cells were cultured as 3-D spheroids in media, high concentrations of IL-6 and TNF-α were found in the spent media and cell adhesion to E-selectin surfaces were found to be dependent on IL-6 concentration.

**Figure 6:**
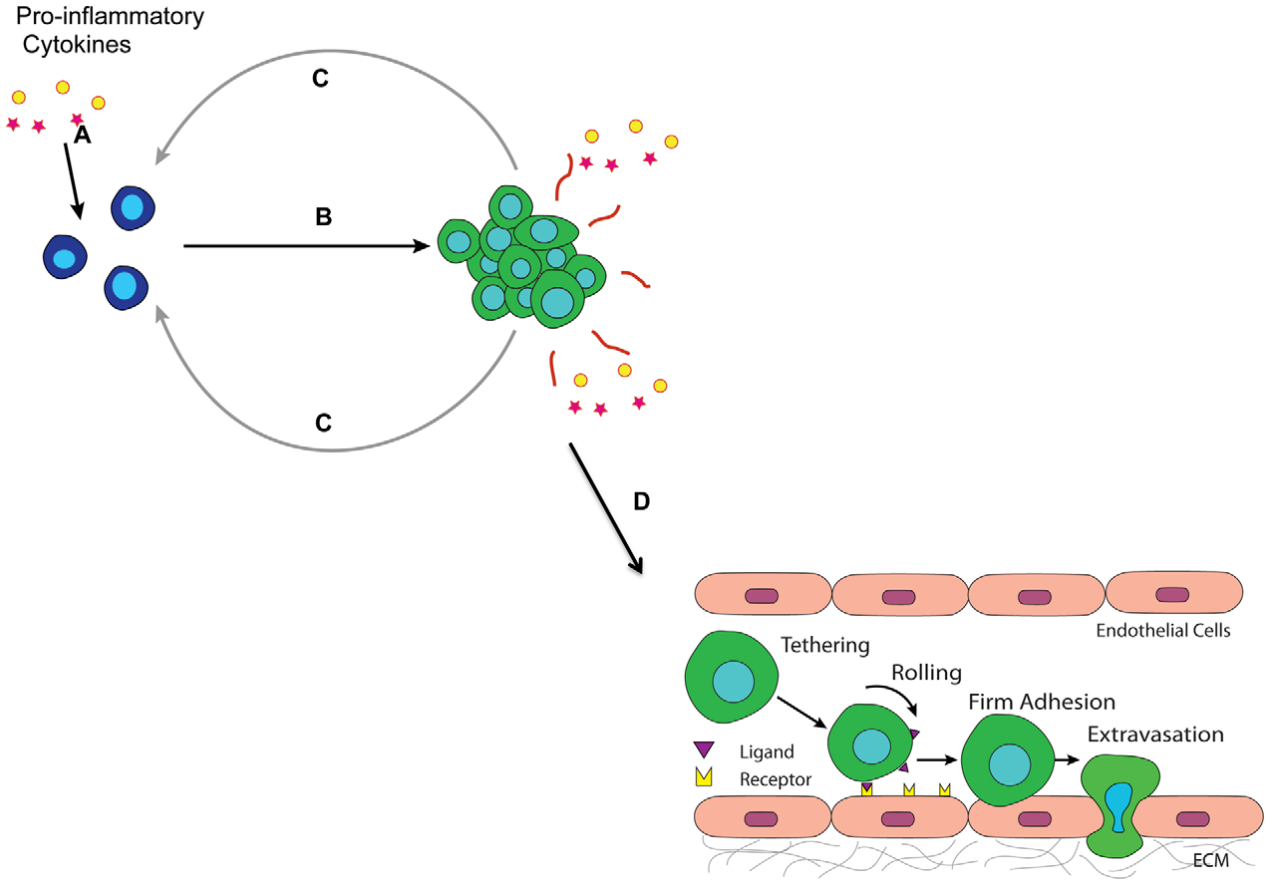
A positive feedback loop: activation and maintenance of the adhesive phenotypic switch. Step A: Tumor cells encounter proinflammatory cytokines such as IL-6 and TNF-α secreted by tumor promoting immune cells in the microenvironment. Step B: Cytokine conditioned tumor cells form aggregates. Step C: Aggregates/spheroids of tumor cells release more IL-6 and TNF-α, turning on a phenotypic switch for more tumor cells nearby by upregulating E-selectin ligand and binding moiety expressions, and promote heterotypic interactions between tumor cells and the inflamed endothelium. Step D: Tumor cells ready to invade the endothelium.

We propose a novel mechanism (Figure 6) that may explain the aggressiveness of ‘triple-negative’ breast carcinoma. First, the pro-inflammatory cytokines act on single cells to induce an aggregated morphology and an increased proliferation rate. Cell aggregation then upregulates the secretion rate of IL-6 and TNF-α thereby increasing cytokine concentrations. This may cause a positive feedback mechanism where the increased cytokine concentrations cause more aggregation and proliferation that further stimulate cytokine secretion. The ultimate effect of aggregation is the increased expression of E-selectin ligands and binding moieties that facilitate stable rolling on the inflamed endothelium to enable subsequent extravasation.

In summary, our results indicate that plasma and cytokines can promote homotypic aggregation and that cell proliferation as tumor spheroids can change the adhesion phenotype of cancer cells to immobilized E-selectin under physiological flow, which may contribute to the higher metastatic potential of CTM. Taken together, these results suggest that therapeutic approaches targeting cytokine receptors and adhesion molecules on cancer cells may help reduce the likelihood of metastasis and deserve further attention.

## Materials and Methods

### Reagents

Recombinant E-selectin-IgG_1_ chimera was purchased from R&D systems (Minneapolis, MN). Blotting grade blocker non-fat dry milk was obtained from Bio-Rad Laboratories (Hercules, CA) and Protein-G was purchased from EMD Biosciences (San Diego, CA). FITC Mouse IgG1 k isotype control, purified mouse anti-human CD15s (clone CSLEX), and APC rat anti-mouse IgM were all purchased from BD Biosciences (San Jose, CA). FITC mouse anti-human CD44V4 was obtained from AbD Serote (Germany). Ca^2+^ and Mg^2+^ free DPBS (Invitrogen, Camarillo, CA, USA), calcium carbonate (Sigma Chemical Co., St. Louis, MO, USA), low endotoxin (1 ng/mg), and essentially globulin-free Bovine Serum Albumin (Sigma Chemical Co., St. Louis, MO, USA) were used to prepare flow buffer for cell adhesion assays. Nuclear ID™ cell viability reagent was obtained from Enzo Life Sciences (Farmingdale, NY).

### Breast Cancer Cell Culture

Breast cancer cell line MDA-MB-231 was purchased from ATCC and maintained in 10% Fetal Bovine Serum (FBS; Cellgro), 1% penicillin-streptomycin (Invitrogen), and high glucose Dulbecco's Modified Eagle Medium at 37°C with 5% CO_2_ in a humidified incubator.

### Plasma isolation and treatment

Whole peripheral blood was collected from informed consenting healthy female donors using sodium–heparin tubes with a venoject vacuum system. Collected whole blood was centrifuged for 25 min at 500 rpm. The plasma layer on top was carefully removed without disturbing the interface and passed through a sterile 0.2 μm filter. 50% of the isolated plasma was used for cell culture while the rest was stored in −80°C for other assays. MDA-MB-231 cells were plated at 2×10^5^ cells/well in 6-well plates. Isolated plasma and culture medium at a 1∶1 ratio were used to treat cells for 48 h.

### Tumor spheroid generation using PDMS coated plate

*Dow Corning*'s Sylgard® 184 silicone elastomer kit in a 10∶1 base to curing agent ratio (w/w) was used to cure PDMS [46]. The pre-polymer components were manually mixed with a pipette tip in a 50 mL tube for 60 s. 300 µL of PDMS pre-polymer was pipetted into each well of a 24-well plate and allowed to settle at room temperature (RT) for 30 min. The plates were then cured at 60°C for 4 h. MDA-MB-231 cells were plated at 5×10^4^ cells/well and cultured for 48 h.

### Pro-inflammatory cytokine and Metformin treatment

Human recombinant IL-6 (R&D) and TNF-α (R&D) were added to the culture media at a range of concentrations from 1–25 ng/mL. Cell rolling assay and flow cytometry were performed after 48 hr treatments (single cytokine or combined). For blocking/neutralization experiments, 0.1 mM Metformin (1,1-dimethylbiguanide hydrochloride) or 1 μg/mL of mouse anti-human IL-6R monoclonal antibody was added with the IL-6 treatment.

### Neuraminidase treatment

After IL-6 treatment and prior to rolling experiments, MDA-MB-231 cells were treated with 0.1 U/ml Vibrio Cholerae neuraminidase (Roche Biochemicals, Indianapolis, IN) for 45 min at 37°C to cleave terminal sialic acid residues. After enzyme treatment cells were washed and incubated with 0.1% BSA to block nonspecific interactions.

### MTT Assay

MDA cells were seeded into each well of a 24-well culture plate. After 12 hr in culture, the medium was withdrawn and replaced with 0.5 mL fresh DMEM culture medium or cytokine spiked medium. After 2 days in culture, all media was withdrawn and replaced with 0.5 mL fresh DMEM medium. Next, 50 μl MTT stock solution (5 mg MTT powder/1 ml PBS) was added into each well and incubated at 37°C for 1 hr. Media was then removed and replaced with 0.5 mL DMSO for 5 min incubation at RT. Absorbance was measured at a wavelength of 570 nm with background subtraction at 660 nm.

### Cell Viability Assay

The Nuclear-ID™ Blue/Red cell viability reagent (GFP-Certified™), a mixture of a blue fluorescent cell-permeable nucleic acid dye and a red fluorescent cell-impermeable nucleic acid dye that is suited for staining dead nuclei was used to determine cell viability. MDA-MB-231 cells with various treatments including control, plasma, IL-6, IL-6 + Metformin, and TNF-α were grown in imaging ready glass coverslip mounted 24-well plates at 5×10^4^ cells/well. Cells were incubated with the Nuclear-ID reagent at 1∶1000 dilution for 30 min at 37°C and imaged with a Zeiss 710 confocal microscope (10X objective) with a dual filter set for DAPI (Ex/Em: 350/470 nm) and Texas Red (Ex/Em: 540/605 nm).

### Flow cytometry

After 48 h in culture, untreated and treated (plasma, tumor spheroid, IL-6, and TNF-α) MDA-MB-231 cells were removed from culture plates using an enzyme-free cell dissociation buffer solution to preserve membrane proteins and prevent clumping. After washing with PBS, the cells were resuspended in 1% BSA in DPBS to a final concentration of 250,000 cells per sample. Antibodies against CD44V4 and sLe^x^ or appropriate isotype controls were added to the cell suspensions and incubated over ice for 45 min. Following incubation, cells were washed twice with 500 μL of 1% BSA to remove any unbound antibody. Flow cytometry samples were analyzed using an Accuri C6 flow cytometer (BD Bioscience, San Jose, CA).

### Cell Rolling Assay

Micro-renathane tubing with 640 μm internal diameter (Braintree Scientific) was cut to a length of 50 cm, functionalized with Protein G (10 μg/mL) and Fc chimera E-selectin (20 μg/mL, R&D), and blocked with 5% BSA or milk (Sigma). Functionalized microtubes were then secured to the stage of an Olympus IX81 motorized inverted microscope (Olympus America, Melville, NY). A CCD camera (model no: KP-M1AN, Hitachi, Tokyo, Japan) and DVD recorder (model no: DVD-1000MD, Sony Electronics) were used to record experiments for offline analysis. MDA-MB-231 cells were suspended in flow buffer at 1×10^6^ cells/mL and perfused through protein coated microtubes using a syringe pump (KDS 230, IITC Life Science, Woodland Hills, CA) at a wall shear stress of 1.0 dyn/cm^2^. “Rolling” cells were defined as those observed to translate in the direction of flow with an average velocity less than 50% of the calculated hydrodynamic free-stream velocity [47].

### Quantification of cytokine concentration using enzyme-linked immunosorbent assay (ELISA)

Healthy donor plasma was isolated as described above. Conditioned media was harvested from both 2-D culture and 3-D tumor spheroid culture. Anti-human IL-6 and TNF-α ELISA kits from eBioscience (San Diego, CA) were used to quantify the cytokine concentration. Standard curves were performed per instruction and the concentration of cytokines in each sample was calculated accordingly.

### Real-time quantitative PCR (qPCR)

10 ng of cDNA produced by the reverse transcription of total RNA was used in each quantitative PCR reaction. Also included in the 20 µL qPCR reaction system were 10 µL iQ^TM^ SYBR Supermix (Bio-Rad), 1 µL of 2 µM forward primer and 1 µL of 2 µM reverse primer and nuclease free water.

### Primer for CD44 qPCR

5′-TATAAGCTTTTCGCTCCGGACACCAT-3′ (Forward)

5′-ATAAGATCTTTCTGGAATTTGGGGTG-3′ (Reverse).

### Primer for GAPDH qPCR

5′-AGAGCACAAGAGGAAGAGAGAGAC-3′ (Forward)

5′-AGCACAGGGTACTTTATTGATGGT-3′ (Reverse).

qPCR reactions were carried out in 96 well real-time PCR plates (Bio-Rad) using a Bio-Rad MyIQ Real-time PCR detection system. The qPCR reaction consisted of 5 min at 95°C to activate the polymerase and 50 PCR cycles (uncoupling step at 95°C for 20 sec followed by annealing step at 59°C for 20 sec and elongation step at 72°C for 30 sec), followed by a melting temperature analysis to test for any nonspecific amplification. The expression level of CD44 gene in MDA-MB-231 cells with different treatment was normalized to the expression level of the standard gene GAPDH.

## References

[1] Stockler M, Wilcken NR, Ghersi D, Simes RJ (2000) Systematic reviews of chemotherapy and endocrine therapy in metastatic breast cancer. Cancer Treat Rev 26: 151–168. 10814559 10.1053/ctrv.1999.0161

[2] Ramaswamy S, Ross KN, Lander ES, Golub TR (2003) A molecular signature of metastasis in primary solid tumors. Nat Genet 33: 49–54. 12469122 10.1038/ng1060

[3] Poste G, Fidler IJ (1980) The pathogenesis of cancer metastasis. Nature 283: 139–146. 6985715 10.1038/283139a0

[4] Pandit TS, Kennette W, Mackenzie L, Zhang G, Al-Katib W, et al (2009) Lymphatic metastasis of breast cancer cells is associated with differential gene expression profiles that predict cancer stem cell-like properties and the ability to survive, establish and grow in a foreign environment. Int J Oncol 35: 297–308. 19578743

[5] Geng Y, Marshall JR, King MR (2012) Glycomechanics of the metastatic cascade: tumor cell-endothelial cell interactions in the circulation. Ann Biomed Eng 40: 790–805. 22101756 10.1007/s10439-011-0463-6

[6] Thorlacius H, Prieto J, Raud J, Gautam N, Patarroyo M, et al (1997) Tumor cell arrest in the microcirculation: lack of evidence for a leukocyte-like rolling adhesive interaction with vascular endothelium in vivo. Clin Immunol Immunopathol 83: 68–76. 9073538 10.1006/clin.1996.4325

[7] Geng Y, Narasipura S, Seigel GM, King MR (2010) Vascular Recruitment of Human Retinoblastoma Cells by Multi-Cellular Adhesive Interactions with Circulating Leukocytes. Cellular and Molecular Bioengineering 3: 361–368. 25110524 10.1007/s12195-010-0145-8 PMC4125030

[8] Yin XY, Rana K, Ponmudi V, King MR (2010) Knockdown of fucosyltransferase III disrupts the adhesion of circulating cancer cells to E-selectin without affecting hematopoietic cell adhesion. Carbohydrate Research 345: 2334–2342. 20833389 10.1016/j.carres.2010.07.028 PMC2995892

[9] Goetz DJ, ElSabban ME, Hammer DA, Pauli BU (1996) Lu-ECAM-1-mediated adhesion of melanoma cells to endothelium under conditions of flow. International Journal of Cancer 65: 192–199. 8567116 10.1002/(SICI)1097-0215(19960117)65:2<192::AID-IJC11>3.0.CO;2-G

[10] Kim YJ, Borsig L, Han HL, Varki NM, Varki A (1999) Distinct selectin ligands on colon carcinoma mucins can mediate pathological interactions among platelets, leukocytes, and endothelium. American Journal of Pathology 155: 461–472. 10433939 10.1016/S0002-9440(10)65142-5 PMC1866847

[11] Kozlowski L, Zakrzewska I, Tokajuk P, Wojtukiewicz MZ (2003) Concentration of interleukin-6 (IL-6), interleukin-8 (IL-8) and interleukin-10 (IL-10) in blood serum of breast cancer patients. Roczniki Akademii Medycznej w Bialymstoku (1995) 48: 82–84. 14737948

[12] Berberoglu U, Yildirim E, Celen O (2004) Serum levels of tumor necrosis factor alpha correlate with response to neoadjuvant chemotherapy in locally advanced breast cancer. International Journal of Biological Markers 19: 130–134. 15255545 10.1177/172460080401900207

[13] Bates RC, Mercurio AM (2003) Tumor Necrosis Factor-α Stimulates the Epithelial-to-Mesenchymal Transition of Human Colonic Organoids. Molecular Biology of the Cell 14: 1790–1800. 12802055 10.1091/mbc.E02-09-0583 PMC165077

[14] Lee SO, Lou W, Hou M, de Miguel F, Gerber L, et al (2003) Interleukin-6 promotes androgen-independent growth in LNCaP human prostate cancer cells. Clin Cancer Res 9: 370–376. 12538490

[15] Muller A, Homey B, Soto H, Ge N, Catron D, et al (2001) Involvement of chemokine receptors in breast cancer metastasis. Nature 410: 50–56. 11242036 10.1038/35065016

[16] Zen K, Liu D-Q, Guo Y-L, Wang C, Shan J, et al (2008) CD44v4 Is a Major E-Selectin Ligand that Mediates Breast Cancer Cell Transendothelial Migration. PLoS ONE 3: e1826. 18350162 10.1371/journal.pone.0001826 PMC2265551

[17] Narita T, Kawakami-Kimura N, Matsuura N, Funahashi H, Kannagi R (1996) Adhesion of human breast cancer cells to vascular endothelium mediated by Sialyl Lewis<sup>x</sup>/E-selectin. Breast Cancer 3: 19–23. 11091549 10.1007/BF02966958

[18] Minn AJ, Kang YB, Serganova I, Gupta GP, Giri DD, et al (2005) Distinct organ-specific metastatic potential of individual breast cancer cells and primary tumors. Journal of Clinical Investigation 115: 44–55. 15630443 10.1172/JCI22320 PMC539194

[19] Witz IP (2008) The selectin-selectin ligand axis in tumor progression. Cancer Metastasis Rev 27: 19–30. 18180878 10.1007/s10555-007-9101-z

[20] Laubli H, Borsig L (2010) Selectins promote tumor metastasis. Seminars in Cancer Biology 20: 169–177. 20452433 10.1016/j.semcancer.2010.04.005

[21] Deng XS, Wang S, Deng A, Liu B, Edgerton SM, et al (2012) Metformin targets Stat3 to inhibit cell growth and induce apoptosis in triple-negative breast cancers. Cell Cycle 11: 367–376. 22189713 10.4161/cc.11.2.18813

[22] Luque-Ramirez M, Escobar-Morreale HF (2010) Treatment of polycystic ovary syndrome (PCOS) with metformin ameliorates insulin resistance in parallel with the decrease of serum interleukin-6 concentrations. Horm Metab Res 42: 815–820. 20730705 10.1055/s-0030-1262855

[23] Fidler IJ (1973) The relationship of embolic homogeneity, number, size and viability to the incidence of experimental metastasis. European Journal of Cancer (1965) 9: 223–227. 10.1016/s0014-2964(73)80022-2 4787857

[24] Glinsky GV, Glinsky VV (1996) Apoptosis and metastasis: a superior resistance of metastatic cancer cells to programmed cell death. Cancer Letters 101: 43–51. 8625281 10.1016/0304-3835(96)04112-2

[25] Coussens LM, Werb Z (2002) Inflammation and cancer. Nature 420: 860–867. 12490959 10.1038/nature01322 PMC2803035

[26] Grivennikov SI, Greten FR, Karin M (2010) Immunity, Inflammation, and Cancer. Cell 140: 883–899. 20303878 10.1016/j.cell.2010.01.025 PMC2866629

[27] Condeelis J, Pollard JW (2006) Macrophages: obligate partners for tumor cell migration, invasion, and metastasis. Cell 124: 263–266. 16439202 10.1016/j.cell.2006.01.007

[28] Ben-Baruch A Host microenvironment in breast cancer development: inflammatory cells, cytokines and chemokines in breast cancer progression: reciprocal tumor-microenvironment interactions. 10.1186/bcr554 PMC154133 12559043

[29] Lyon DE, McCain NL, Walter J, Schubert C (2008) Cytokine comparisons between women with breast cancer and women with a negative breast biopsy. Nurs Res 57: 51–58. 18091292 10.1097/01.NNR.0000280655.58266.6c PMC2234268

[30] Lehmann BD, Bauer JA, Chen X, Sanders ME, Chakravarthy AB, et al (2011) Identification of human triple-negative breast cancer subtypes and preclinical models for selection of targeted therapies. J Clin Invest 121: 2750–2767. 21633166 10.1172/JCI45014 PMC3127435

[31] Marui N, Offermann MK, Swerlick R, Kunsch C, Rosen CA, et al (1993) Vascular cell adhesion molecule-1 (VCAM-1) gene transcription and expression are regulated through an antioxidant-sensitive mechanism in human vascular endothelial cells. The Journal of Clinical Investigation 92: 1866–1874. 7691889 10.1172/JCI116778 PMC288351

[32] Lagow EL, Carson DD (2002) Synergistic stimulation of MUC1 expression in normal breast epithelia and breast cancer cells by interferon-gamma and tumor necrosis factor-alpha. J Cell Biochem 86: 759–772. 12210742 10.1002/jcb.10261

[33] Zhang P, Ozdemir T, Chung CY, Robertson GP, Dong C (2011) Sequential binding of αVβ3 and ICAM-1 determines fibrin-mediated melanoma capture and stable adhesion to CD11b/CD18 on neutrophils. J Immunol 186: 242–254. 21135163 10.4049/jimmunol.1000494 PMC3058329

[34] Sullivan NJ, Sasser AK, Axel AE, Vesuna F, Raman V, et al (2009) Interleukin-6 induces an epithelial-mesenchymal transition phenotype in human breast cancer cells. Oncogene 28: 2940–2947. 19581928 10.1038/onc.2009.180 PMC5576031

[35] Muthukumaran N, Miletti-Gonzalez KE, Ravindranath AK, Rodriguez-Rodriguez L (2006) Tumor necrosis factor-alpha differentially modulates CD44 expression in ovarian cancer cells. Mol Cancer Res 4: 511–520. 16908592 10.1158/1541-7786.MCR-05-0232

[36] Davril M, Degroote S, Humbert P, Galabert C, Dumur V, et al (1999) The sialylation of bronchial mucins secreted by patients suffering from cystic fibrosis or from chronic bronchitis is related to the severity of airway infection. Glycobiology 9: 311–321. 10024669 10.1093/glycob/9.3.311

[37] Groux-degroote S, Krzewinski-recchi M-A, Cazet A, Vincent A, Lehoux S, et al (2008) IL-6 and IL-8 increase the expression of glycosyltransferases and sulfotransferases involved in the biosynthesis of sialylated and/or sulfated Lewisx epitopes in the human bronchial mucosa. Biochem J 410: 213–223. 17944600 10.1042/BJ20070958

[38] Geng Y, Yeh K, Takatani T, King MR (2012) Three to Tango: MUC1 as a Ligand for Both E-Selectin and ICAM-1 in the Breast Cancer Metastatic Cascade. Front Oncol 2: 76. 22866263 10.3389/fonc.2012.00076 PMC3406322

[39] Hughes AD, Mattison J, Western LT, Powderly JD, Greene BT, et al (2012) Microtube device for selectin-mediated capture of viable circulating tumor cells from blood. Clin Chem 58: 846–853. 22344286 10.1373/clinchem.2011.176669

[40] Hughes AD, Mattison J, Powderly JD, Greene BT, King MR (2012) Rapid Isolation of Viable Circulating Tumor Cells from Patient Blood Samples. J Vis Exp: e4248. 10.3791/4248 PMC3471307 22733259

[41] Agastin S, Giang UBT, Geng Y, DeLouise LA, King MR (2011) Continuously perfused microbubble array for 3D tumor spheroid model. Biomicrofluidics 5. 10.1063/1.3596530 PMC3124519 21716809

[42] Hou J-M, Krebs M, Ward T, Morris K, Sloane R, et al (2010) Circulating Tumor Cells, Enumeration and Beyond. Cancers 2: 1236–1250. 24281115 10.3390/cancers2021236 PMC3835128

[43] Paterlini-Brechot P, Benali NL (2007) Circulating tumor cells (CTC) detection: Clinical impact and future directions. Cancer Letters 253: 180–204. 17314005 10.1016/j.canlet.2006.12.014

[44] Hirschhaeuser F, Menne H, Dittfeld C, West J, Mueller-Klieser W, et al (2010) Multicellular tumor spheroids: An underestimated tool is catching up again. Journal of Biotechnology 148: 3–15. 20097238 10.1016/j.jbiotec.2010.01.012

[45] Chandrasekaran S, Geng Y, Delouise LA, King MR (2012) Effect of homotypic and heterotypic interaction in 3D on the E-selectin mediated adhesive properties of breast cancer cell lines. Biomaterials 33: 9037–9048. 22992472 10.1016/j.biomaterials.2012.08.052 PMC3515853

[46] Chandrasekaran S, Giang UB, King MR, DeLouise LA (2011) Microenvironment induced spheroid to sheeting transition of immortalized human keratinocytes (HaCaT) cultured in microbubbles formed in polydimethylsiloxane. Biomaterials 32: 7159–7168. 21724250 10.1016/j.biomaterials.2011.06.013 PMC3148275

[47] Lee D, Schultz JB, Knauf PA, King MR (2007) Mechanical shedding of L-selectin from the neutrophil surface during rolling on sialyl Lewis x under flow. Journal of Biological Chemistry 282: 4812–4820. 17172469 10.1074/jbc.M609994200

